# Visual training program for body dysmorphic disorder: protocol for a novel intervention pilot and feasibility trial

**DOI:** 10.1186/s40814-018-0384-3

**Published:** 2018-12-21

**Authors:** Francesca Beilharz, David J. Castle, Andrea Phillipou, Susan L. Rossell

**Affiliations:** 1 Centre for Mental Health, Swinburne University of Technology, Melbourne, Australia; 2 Department of Psychiatry, St Vincent’s Hospital, Melbourne, Australia; 3 Psychiatry, Faculty of Medicine, University of Melbourne, Melbourne, Australia

**Keywords:** Body dysmorphic disorder, Visual training, Cognitive behavioural therapy, Cognitive remediation therapy, Feasibility, Pilot trial

## Abstract

**Background:**

Body dysmorphic disorder (BDD) is a characterised by perceived defects or flaws in appearance which are associated with distressing thoughts, repetitive or obsessive behaviours, and significant impairment in social and occupational functioning. A core feature of BDD involves abnormalities of visual processing, although this is not typically a focus of psychological and psychiatric treatments. While current treatments generally show moderate effectiveness in the short-term, those with BDD can have high relapse rates, as they still ‘see’ their flaws or defects. The current research trials a visual training program designed to remediate visual abnormalities and reduce symptom severity of BDD.

**Methods:**

This is a single-group open-label pilot study assessing the feasibility and potential efficacy of a 10-week visual training program. This pilot trial will be conducted at Swinburne University of Technology, Melbourne, Australia, and will recruit up to 20 participants diagnosed with BDD. These participants will complete pre- and post-assessments and a 10-week visual training program encompassing three phases of basic visual processing, face and emotion recognition, and self-perception. The primary outcomes focus on feasibility and acceptability of the intervention, with secondary outcomes exploring clinical outcomes related to symptom severity, quality of life and eye movements.

**Discussion:**

This pilot trial will translate the empirical findings of abnormalities in visual processing among those diagnosed with BDD, to an innovative treatment method across a range of visual processing levels. This trial will assess the feasibility and potential efficacy of such a visual training program, paving the way for further research including a future definitive randomised control trial.

**Trial registration:**

Australian New Zealand Clinical Trial Registry, ACTRN 12618000274279, Registered 22nd February 2018.

**Electronic supplementary material:**

The online version of this article (10.1186/s40814-018-0384-3) contains supplementary material, which is available to authorized users.

## Background

Body dysmorphic disorder (BDD) is a psychiatric disorder characterised by a preoccupation with perceived flaws or defects in physical appearance, which are associated with distressing thoughts, obsessive or repetitive behaviours and significant impairment in daily functioning [1]. BDD affects approximately 1.9% of the general population, with slightly higher rates among women in most settings [1, 2]. Individuals diagnosed with BDD often have concerns regarding multiple aspects of their appearance, although common areas include specific facial features (e.g. nose), texture or colour of hair and skin, or specific body parts (e.g. thighs) [3]. If individuals are predominantly concerned with overall body weight or shape, it should be considered whether these symptoms are better explained by an eating disorder [1]. Comorbid disorders are common accompaniments of BDD, including depression, anxiety, social anxiety, obsessive-compulsive disorder and substance abuse [4–6]. Individuals with BDD have suicide rates up to 45 times higher than the general population, and are often housebound [7, 8].

Despite the significant impact BDD can have upon the wellbeing and daily functioning of those who experience it, this disorder is significantly under-researched compared to many other mental health disorders. Consequently, relatively little is understood regarding how symptoms are initiated, maintained and treated. Current guidelines recommend treating BDD with cognitive behavioural therapy (CBT), selective serotonin reuptake inhibitors (SSRIs) or a combination of these psychological and psychiatric treatments [9]. Such treatments generally show moderate effectiveness rates for BDD in the short-term, although symptoms can remain in the mild-moderate severity range at long-term follow-up [10–12]. It is possible that BDD has such high relapse rates compared to many other mental health disorders as current treatments do not address a core feature of the disorder: visual perception. For example, a general aim of CBT for BDD may be to analyse one’s thoughts, emotions and behaviours relating to appearance concerns, but this does not necessarily remove the client’s perception of their ‘flaw’ or ‘abnormalities’ in physical appearance.

Innovative treatment methods may target this gap in the knowledge base by translating empirical research of visual abnormalities within BDD into clinical practice. It is hypothesised that visual processing abnormalities may maintain symptoms of BDD by influencing mirror checking or avoidance behaviours, which reinforce patterns of rumination, avoidance and safety behaviours [13]. Research has demonstrated that people with BDD demonstrate differences in how they perceive images compared to healthy controls reflecting clinical characteristics of the disorder [14]. These abnormalities have been demonstrated for a range of appearance and non-appearance related stimuli, indicating more widespread differences in perception than simply the processing of self. A major theory explaining such differences in visual processing involves the dysregulation of the local and global processing systems in BDD [15]. In healthy individuals, a combination of local (piecing together specific features) and global (overall shape and form) strategies are used to recognise and identify visual stimuli [16, 17]. However, those with BDD demonstrate a bias for processing visual information in a local manner, often at the expense of perceiving the overall image. This bias is demonstrated using the face inversion effect, a task where healthy individuals struggle as their global template for faces is disrupted by inverting the face stimulus 180°. People with BDD are thus generally faster and more accurate at this task, as their local bias assists them [18–20].

Reflecting this local bias, eye-tracking studies have demonstrated that individuals with BDD can use aberrant scan paths when viewing stimuli such as faces. These scan paths have generally followed either a ‘focused’ or ‘avoidant’ pattern, by focusing attention on areas of concern, or avoiding perceived flaws, respectively [21–23]. BDD participants displayed additional disruptions in their eye movements, including significantly more blinks, fewer and longer fixations (“hypo-scanning”) and less attention paid to salient facial features. Such difficulties in processing faces appear particularly prominent in BDD when viewing images of themselves and faces displaying negative or neutral emotional expressions [22, 23].

While eye-tracking studies have examined the visual scan paths used by BDD participants to process face stimuli, to date, no published studies have investigated how those with BDD perform on more ‘basic’ saccade tasks. Such simple tasks can assess stable abnormalities in eye movement parameters, which can reflect biomarkers of psychological disorders as demonstrated in anorexia nervosa [24, 25] and schizophrenia [26–28]. As part of the broader project encapsulating this trial, the authors are in the process of conducting an empirical study comparing BDD and healthy control participants’ performance on a battery of eye-tracking tasks, the results of which will inform this trial.

This local bias in visual processing by people with BDD, as identified by differences in accuracy and reaction time scores with unhelpful scan paths, appears to extend to other visual stimuli, including houses, other objects, gestalt shapes, and disorder-related words [15, 19, 29–31]. Neuroimaging results have also supported similar findings, with reduced activity in primary and secondary visual cortices present for a range of appearance and non-appearance related stimuli [29, 32, 33]. BDD participants have displayed reductions in brain volume in areas associated with processing face stimuli: the inferior frontal gyrus and right amygdala, with greater reductions occurring with greater symptom severity [34–36]. In addition, more widespread connectivity issues have been demonstrated between all four lobes in the brain among BDD participants, particularly when transferring visual information across hemispheres [32, 37, 38]. These findings support significantly disrupted white matter connectivity, which implicates difficulties in efficiently processing global information.

Despite these visual abnormalities comprising a core component of the disorder, only one published study to date has attempted to alter visual perception in BDD. Using an emotion recognition program, Buhlmann, Gleiss [39] demonstrated that BDD participants who were provided with correctional feedback showed significant improvements in recognising neutral and scared face stimuli, a well-documented visual processing deficit in BDD [22, 23, 40–42]. However, this visual training research targeted only a single aspect of visual perception, while the literature suggests abnormalities that are more widespread. It has therefore been suggested that a broader and more comprehensive visual training program should be implemented with BDD participants, in an attempt to remediate visual abnormalities and reduce symptom severity [10, 43, 44].

To address this, the authors have designed a visual training program based on a combination of cognitive remediation computer programs utilised in other psychiatric disorders and psychological strategies drawn from cognitive behaviour therapy for BDD. This program has been designed in a ‘bottom-up’ method, to address differences in visual perception evidenced by the literature, in a systematic and additive manner. Activities from the Posit Science system will be implemented for the first two phases of the program (basic visual training and face/emotion recognition), which have been successfully utilised in a variety of psychiatric and neurological populations [45–55]. Posit Science consists of a series of ‘brain training’ activities reflecting a range of cognitive deficits or weaknesses, which have been developed by a team of neuroscientists led by Dr. Merzenich [56]. Posit Science is the only program which meets all requirements of the Institute of Medicine’s Checklist for Brain Training [57]. The mirror retraining phase was combined with these computer remediation techniques to extend visual training to self-perception, using the dominant self-perception technique from CBT for BDD in the literature [58]. The specific phases and activities which were combined to create this novel visual training program were chosen by the authors in collaboration with specialists in the field and based on the existing research literature.

### Study goals and objectives

#### Aims

This project will assess the feasibility of a novel intervention method for BDD by attempting to remediate documented abnormalities in visual perception. This visual training program involves a combination of computer remediation and therapist-led cognitive behavioural therapy tasks, involving the three phases of basic visual processing, face and emotion recognition and self-perception using mirror retraining. This program will be conducted with up to 20 BDD participants over 10 weekly sessions, with comprehensive pre- and post-assessments, and brief assessments of symptom severity and eye movement performance at weeks 4, 8, and 10.

In this trial, the research aim was to explore trial design, participant acceptability of the intervention and feasibility of delivery. The primary objectives are below:

1. To assess recruitment rates
2. To assess retention rates of participants across the 10-week trial
3. To investigate the acceptability of the visual training program among BDD participants in terms of compliance with the proposed schedule, adverse events and qualitative feedback

The secondary objectives of the trial are to:

1. To assess key clinical outcomes (e.g. symptom severity, quality of life and eye movement results) for completion rates, missing data, estimates, variances and potential efficacy using quantitative measures
2. To collect and synthesise data to estimate the sample size and analysis required of a definitive RCT
3. To establish suitable procedures (and factors influencing this) for conducting the assessments and delivering the intervention to ensure successful recruitment and retention within this clinical population

## Methods

### Design

**Fig. 1:**
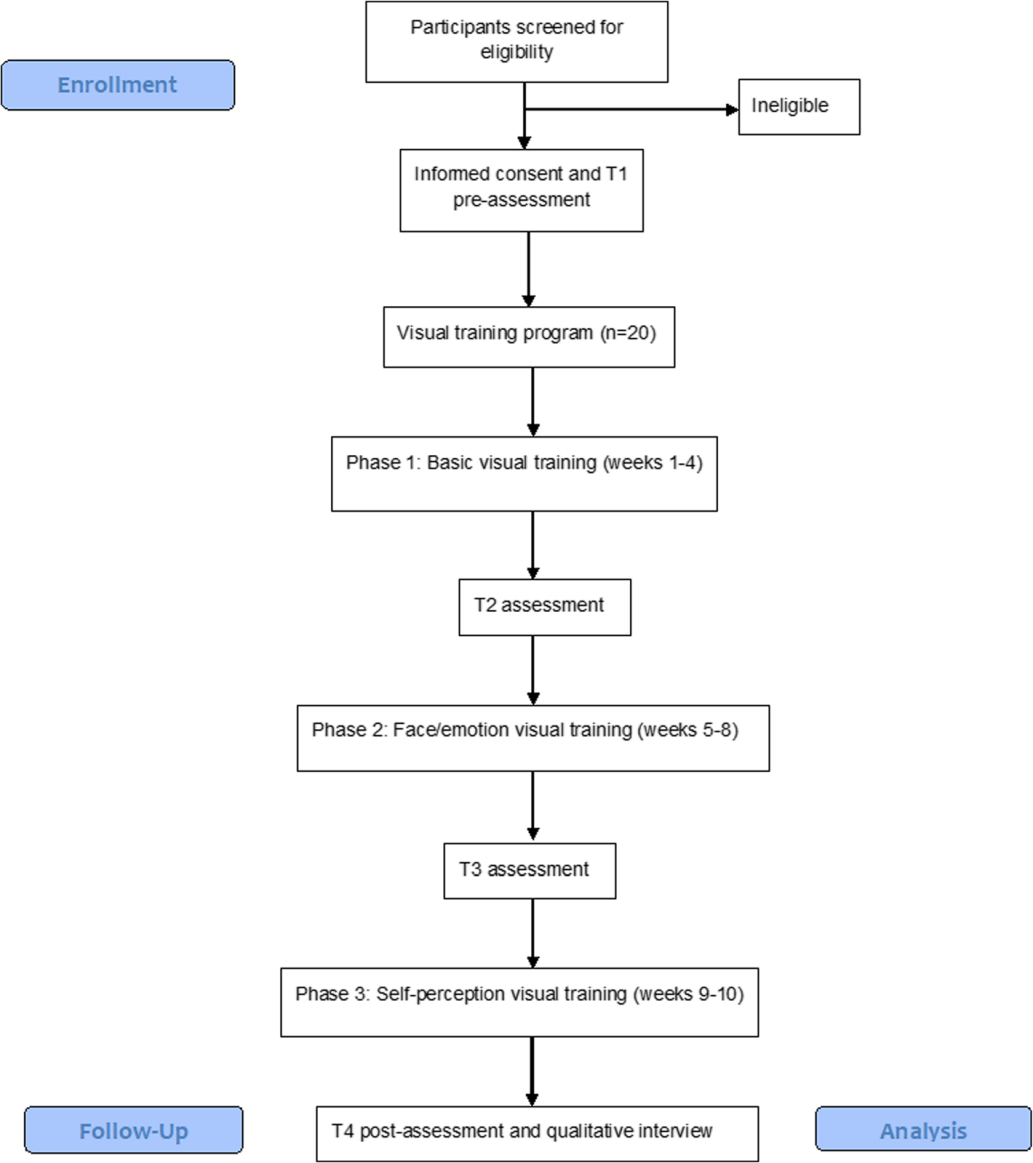
CONSORT flow diagram

Due to the innovative nature of this visual training program, this research will employ a single-group pre-post pilot study design. All recruited participants will take part in the visual training program, with baseline assessments conducted at T1 and T4 after the 10-week program has been completed. All participants will be invited to complete the outcome assessment regardless if they complete the 10-week program or not. Participants will also be asked to complete change assessments at T2 (4 weeks) and T3 (8 weeks) to assess for any specific changes on outcome measures after each phase of the trial is complete. Please see Fig. 1 for the Consolidated Standards of Reporting Trials (CONSORT) flow diagram of the study procedure. The present trial was designed in consideration of the CONSORT extension to pilot studies [59]. Table 1 summarises activities and timing of participation, which will result in a total of 16.8 h training over 10 weeks. Participants will be reimbursed with $50 when they complete each of the pre- and post-assessments and will be given $70 to cover costs for travel over the total 10-week training and assessment period.

**Table 1:** Visual training program weekly activities

Schedule	Visual training program activities	Approximate time
Pre	Initial assessment	2 h 30 min (+ break)
Week 1	Basic visual training	2 h (+ 20-min break)
Week 2	Basic visual training	2 h (+ 20-min break)
Week 3	Basic visual training	2 h (+ 20-min break)
Week 4	Basic visual training + change assessment	2 h 15 min (+ 20-min break)
Week 5	Face visual training	2 h (+ 20-min break)
Week 6	Face visual training	2 h (+ 20-min break)
Week 7	Face visual training	2 h (+ 20-min break)
Week 8	Face visual training + change assessment	2 h 15 min (+ 20-min break)
Week 9	Self-perception training	1 h
Week 10	Self-perception training + change assessment	1 h 15 min
Post	Final assessment	2 h 30 min (+ break)

### Participants

Twenty participants with BDD will be recruited through clinical referral pathways with participating clinicians and media advertising and from the Body Image Disorder Participant Registry. Clinicians involved in the ethics application (Prof David Castle, Dr. Ben Buchanan and Dr. Ryan Kaplan) will introduce the study to potential participants by providing a flyer. Interested potential participants can then provide consent for the clinician to pass their contact details to FB for screening purposes or they can directly contact FB via information provided in the advertising materials. To facilitate recruitment, study advertisements will also be placed on social media platforms and in community spaces, where interested potential participants may directly contact FB for further information. In addition, participants may be recruited from the Body Image Disorder Participant Registry which is a database of individuals who have provided consent to be contacted for future research projects, which is located at Swinburne University. All recruitment processes have been approved by SUHREC. Contact is then made by FB to interested potential participants via phone to provide a full description of the study, with emphasis on the duration of the trial and requirements of participants. During this phone contact, participants are also screened to ensure inclusion and exclusion criteria are met, as seen below. Eligible and interested participants can then arrange an appointment with FB to provide written informed consent and complete the initial assessment. Participants will be sent the PICF and have the opportunity to ask any questions prior to providing written consent.

Inclusion criteria:

1. Primary current diagnosis of body dysmorphic disorder
2. Aged between 18 and 65 years old
3. Preferred language of English
4. Stable on psychiatric medication or psychological treatment (if any) over the past 8 weeks
5. Participants may be engaged in current psychological or psychiatric treatment either for BDD or comorbid psychological symptoms
6. Able to attend 10 weekly sessions at Swinburne University, Hawthorn, VIC, Australia

Exclusion criteria:

1. Lifetime history of neurological or ocular conditions
2. Lifetime history of anorexia nervosa, bulimia nervosa, substance dependence disorder or psychotic disorder
3. Involvement in any current interventional trial
4. Considered inappropriate by the referring clinician, e.g. motivation, wellbeing

Due to the high prevalence of avoidant behaviours present in BDD, the researchers have considered a number of assisting strategies for recruitment and retention. For example, while Swinburne University is easily accessed via public transport, the authors acknowledge that this method of travel may be particularly distressing for some participants with BDD, such as those with high social anxiety. As such, we have arranged parking access for participants who would prefer to drive, to increase engagement over the course of the trial. The authors also acknowledge that some components of the trial protocol may feel distressing for some participants with BDD to complete, such as having their photograph taken and included in the eye-tracking assessment or participating in the mirror retraining phase. It is important in the study to ascertain these avoidant behaviours present as they may influence feasibility; they will thus be recorded to allow for the research team to make decisions about future management.

### Outcomes

#### Feasibility and acceptability

The primary outcomes of this trial focus on feasibility and acceptability, with secondary outcomes evaluating clinical measures. Feasibility will be assessed in terms of recruitment numbers (success defined as minimum of 10 participants enrolled over a period of 12 months), completion (with completers defined as participants who complete 100% of the proposed intervention) and attrition rates (success defined as less than 20% attrition), adverse events and fidelity to the protocol as defined by protocol violations.

Acceptability of the intervention will be assessed using a specifically designed qualitative interview, to explore participants’ experiences and feedback. A semi-structured interview will be administered which was developed by the research team, including those with experimental and clinical experience working with body dysmorphic disorder (please see Additional file 1). Thirteen questions will be asked, encompassing the following:

1. Overall experience, for example, tell me what you thought about the program?
2. General benefits, for example, what did the program do for you?
3. Stimulus, for example, what tasks did you like the most?
4. Cognitive trainer, for example, what did you think was the role of the provisional psychologist?
5. Structure of session, for example, did you like the way the sessions were run?

This interview will be audio recorded with the participant’s consent. Participants will be given the opportunity to provide any additional feedback not queried in the interview, and the interviewer may clarify any aspects as necessary. Participants will also complete the Helping Alliance Questionnaire (HAQ) to assess the presence of therapeutic factors which may have contributed to the results. The HAQ is a well-validated measure for assessing patients’ perceptions of the quality of the therapeutic alliance [60, 61].The authors established guidelines for specific feasibility and acceptability criteria in determining whether a future definitive RCT may be undertaken. These criteria included (a) the proportion of participants referred/contacted participating in the visual training program would be 50% or greater, (b) the proportion of participants completing the visual training program would be 50% or greater and (c) the qualitative feedback would predominantly reflect participant satisfaction with the visual training program with less than 30% reporting negative perceptions.

#### Clinical outcomes

During the pre- and post-assessments, demographic variables will be collected encompassing gender, age, education, employment, cigarette use and medications. The following measures are used within the pre- and post-assessments for clinical outcomes: clinical interview, self-report questionnaires and eye-tracking tasks. Each assessment will take approximately 2.5 h to complete. All assessments will be administered by trained staff or students in SR’s laboratory to ensure FB who is administering the visual training program will be blind to the pre- and post-assessment results of all participants.

#### Clinical interviews

The Mini Neuropsychiatric Interview (MINI) version 7.0 for DSM-5 and Body Dysmorphic Disorder Module (BDDM) will be used to screen for psychiatric disorders and to confirm primary diagnosis of BDD [62, 63]. The MINI assesses for lifetime and current prevalence of the 17 most common psychiatric disorders, by screening with a yes/no format and ruling out any medical, organic or drug-related causes. As BDD is not assessed in the MINI, the BDDM assesses the presence of BDD symptoms. These semi-structured clinical interviews have been well validated in clinical populations with strong psychometrics, including within BDD samples [62–64]. The Yale-Brown Obsessive-Compulsive Scale- Body Dysmorphic Disorder version (BDD-YBOCS) will be used to assess symptom severity [65]. The 12 items in this measure are rated on a 5-point Likert scale from 0 = absent to 4 = extreme symptoms. The BDD-YBOCS has been well validated in BDD populations and classifies symptoms as ‘mild’ (0–15), ‘moderate’ (16–30) and ‘severe’ (31–48) [65, 66].

#### Self-report questionnaires

A range of self-report measures will be used to assess a range of psychological and physical domains. The Dysmorphic Concern Questionnaire (DCQ) will measure levels of dysmorphic concern indicative of a BDD diagnosis [67]. The seven items are rated on a 4-point Likert scale from 0 = not at all to 3 = much more than most people, with higher scores indicating greater dysmorphic concern. A cutoff score of 9 will be used following literature recommendations [68]. The DCQ is valid and sensitive, with good psychometrics across BDD and general populations [67–69].

The Body Dysmorphic Disorder Questionnaire (BDDQ) will assess symptoms of BDD using five items presented in a yes/no format [63]. Positive answers for four or five items are required for a positive screening to meet clinical criteria. The BDDQ has been well validated in clinical and general populations [63, 70].

The Depression Anxiety Stress Scale (21-item version; DASS-21) will assess the severity of symptoms associated with anxiety and depressive disorders [71]. The 21 items are scored on 3-point Likert scale, from 0 = never to 3 = almost always. Responses have been categorised into mild, moderate and severe [71]. The DASS-21 demonstrates strong psychometrics and is sensitive to the constructs assessed [72].

The Social Interaction Anxiety Scale (SIAS) will assess for symptoms of social anxiety disorder [73]. Twenty items are rated on a 5-point Likert scale from 0 = not at all characteristic of me to 4 = extremely characteristic of me. Total scores range from 0 to 60, with a cut off of 34 recommended to indicate clinical levels of social anxiety [73]. The SIAS demonstrates adequate validity and reliability in the general population and clinical samples [74].

The Body Appreciation Scale (BAS) will be used to assess ‘positive body image’ of participants using a Likert scale from 1 = never to 5 = always across 13 items [75]. Scores are summed and then averaged across five domains, with higher scores indicating greater body appreciation. The BAS has demonstrated good psychometrics [75].

The Mirror Gazing: Cognition and Affect Rating Scale (MG-CARS) will measure appearance-related distress, mirror use and focus of attention [44]. Six items are rated along visual analogue scales and were developed from the CBT model of BDD. Good psychometrics has been demonstrated [44].

The Creative Achievement Questionnaire (CAQ) will measure participants’ sense of creativity compared to others [76]. Ten items in a range of creative domains are rated from 0 = I have no training or experience in this field to 7 = my work has been critiqued in national publications. Validity and reliability have been sufficiently demonstrated [76].

The Difficulties in Emotion Regulation Scale (DERS) will assess multiple aspects of emotion dysregulation [77]. Participants rate 36 items from 1 = almost never to 5 = almost always, giving total scores and subscale scores on nonacceptance, goals, impulses, awareness, strategies and clarity. Higher scores indicate greater difficulty in emotion regulation. Acceptable psychometrics has been established for the DERS [78].

The Assessment of Quality of Life (AQoL-4D) will assess health-related quality of life in the subdomains of independent living, mental health, social relationships and physical senses [79]. The 12 items are scored on a 4-point Likert scale from 1 = I need no help at all to 5 = I need daily help with most tasks. An algorithm is provided by the creators to give a total score ranging from − 0.04 (worse than death) to 1 (full health). The AQoL-4D has strong psychometrics [80].

The Multidimensional Assessment of Interoceptive Awareness (MAIA) will assess bodily sensations and awareness in relation to emotions [81]. A total of 32 items are rated from 0 = never to 5 = always, giving a total score and subscale scores. Higher scores indicate positive levels of interoceptive awareness, with norms in an eating disorder population. The MAIA has demonstrated very good validity and reliability [81].

The Sensory Perception Quotient (SPQ) will assess self-reported visual perception in daily activities [82]. Only the items from the visual subscale will be included, consisting of 20 items rated from 0 = strongly disagree to 3 = strongly agree. Lower scores indicate higher sensory sensitivity. The SPQ and subscales have demonstrated good psychometrics [82].

#### Eye-tracking battery

The eye-tracking tasks will be recorded with the EyeLink1000 Plus system (SR Research, Ontario, Canada), monocularly (left eye) at 1000 Hz. All tasks will be preceded by a calibration sequence. Tasks will be analysed with SR Research’s analysis program ‘DataViewer’ and with custom-made programs in MATLAB.

##### Fixation task

A fixation cross will be presented in the centre of the screen and participants will be required to fixate on it for the entire duration of the task (1 min). This task will be analysed in terms of the rate of ‘saccade intrusions’, namely square wave jerks (pairs of saccades moving the eye away and back to central fixation within 200 ms).

##### Prosaccade task

Target dots will appear at different angles to the left and right (at 5°, 10° and 15°), with pseudorandomised timing and location. Participants will be required to look at the dots when they appear. The task will involve 78 trials and will be analysed for latency (saccadic reaction time to the target stimulus), gain (saccadic amplitude divided by target amplitude giving a measure of saccadic ‘accuracy’) and peak velocity (peak speed of saccades).

##### Antisaccade task

Dots will appear at different angles (5°, 10° and 15°), with pseudorandomised timing and location. Participants will be required to not look at the target when it appears on screen, but to immediately look the same distance from the centre of the monitor but in the opposite direction of where the stimulus appeared. The task will consist of 78 trials and will be analysed in terms of the rate of errors, and the gain, latency and peak velocity of correct responses. A practice task will be conducted first to ensure participants understand the instructions.

##### Memory-guided task

Participants will be asked to fixate on a cross in the centre of the screen while another stimulus is presented briefly in the periphery (50 ms; 5°, 10° or 15° horizontally). The stimulus will disappear after a short period and then the participant will be required to look at (make a saccade towards) where they recall the stimulus in the periphery to have appeared. The task will involve 52 trials and will be analysed in terms of inhibitory error rate (a saccade made to the briefly presented stimulus before being cued to do so), directional error rate (once cued to make a saccade, making a saccade in the opposite direction of the presented stimulus), as well as gain, latency and peak velocity of correct responses and inhibitory errors. Participants will complete a practice task.

##### Facial affect task

Participants will be asked to examine a target black and white photograph showing one of six human models (three females and three male), each displaying one of seven universal emotions. The photographs will be presented for 5 s, with 64 trials. After the photograph is removed, participants are required to identify the shown emotion using a forced-choice paradigm by clicking the mouse “happy”, “sad”, “anger”, “surprise”, “disgust”, “fear” or “neutral”. This task will include a photograph of the participants’ own face (if they consent), displaying a neutral emotion. Participants will initially complete a practice task to ensure they understand the instructions.

##### Body attractiveness task

Participants will be asked to rate the attractiveness of human body stimuli (25 females and 25 males in black clothing). Stimuli will be presented for 5 s, with 50 trials. After each human body is shown, participants are required to click with the mouse on a scale of 1 (least attractive) to 7 (most attractive).

##### Visual search task

Participants will be shown a collection of shapes (red and blue triangles facing different directions) and are required to search for a particular target among the distractor shapes. Specifically, participants are required to search for a red triangle pointing towards the right (target), which may or may not be present in each visual scene. A screen then asks if the target shape was present, and participants can respond by clicking with the mouse on ‘yes’ or ‘no’. Seventy trials of increasing difficulty will be presented, with each presented for 5 s. A practice trial will be conducted first, to ensure participants understand the task.

#### Brief assessment to track change

The assessment to track for any change throughout the three phases of visual training will include a brief symptom severity measure of BDD and a short eye-tracking task. The memory-guided saccade task was chosen as pilot work has indicated that it might be a useful indicator within this population, with inhibitory error rate as the variable of interest. This brief assessment will take approximately 15 min to complete and is composed of the Dysmorphic Concern Questionnaire (DCQ) and Memory-guided eye-tracking task, as described above.

At the beginning of each week’s visual training activities, participants will also complete a brief self-report questionnaire (please see Additional file 2) to assess how much time and effort, if any, they have spent practicing or thinking about the previous weeks training. This will attempt to control for any homework practiced by participants alongside the required visual training program activities.

### Planned interventions

The visual training program will involve three phases of visual training presented in a bottom-up order of processing, based on the literature of visual abnormalities present in BDD. This program will be administered by provisional psychologist FB and involves a grand total of 16.8 h of training (90 min per week), based on the recommendations of general cognitive remediation research [46, 47, 50, 53, 56]. The following tasks from Posit Science will be presented using the ‘Personalised Trainer’ format, where the administrator predetermines certain exercises, but presentation is based on individual performance. The administrator can observe participation and progress to monitor adherence. Participants are free to withdraw from the intervention at any time on their request.

#### Phase 1: Basic visual training

The first phase of the visual training program involves training in basic and general visual principles (such as scanning, attention, movement and details), using the Posit Science (Brain HQ) computer-training module. This phase will be administered in one-on-one face-to-face sessions with provisional psychologist FB who will instruct and support the participants. Specific visual training tasks were selected on the basis of relevant basic visual processes.

Visual sweeps improves overall visual acuity and speeds up visual processing. Participants are required to watch two patterns that ‘sweep’ in or out and identify the direction of the movement by clicking with a mouse. As participants improve, the exercise can change in terms of the speed, colours, direction and thickness of the sweep patterns. Baseline speed presents each sweep for 200 ms and adjusts according to the participant’s accuracy (e.g. speeds up presentation if previous answer was correct). Visual sweeps includes four different stages of colour and direction, with each task consisting of 30 trials. Total score is the presentation time (in milliseconds of the final trial).

Target tracker improves divided attention. Participants are required to keep track of target objects among distractor objects and click on the targets with the mouse when all of the objects stop moving. The exercise increases in difficulty as objects travel faster, the contrast decreases between the objects and background and the exercise adapts to participants’ performance by changing the number of target objects. Target tracker includes four different stages of speed and contract, with each task consisting of 10 trials. Baseline begins with two target objects to track, with additional target objects added or removed according to the participant’s accuracy (e.g. more targets added if previous answer was correct). The total score is the overall number of items tracked accurately.

Eye for detail improves ability to make saccades quickly and to notice subtle details, using visual working memory. Three to five images appear, one at a time, in different positions on the screen. Of the pictures, some match precisely, while others are similar, but not identical. Participants are required to identify where the two identical images appear by clicking on the positions with the mouse. As participants improve, the images flash by more quickly, become more similar and therefore harder to distinguish, are spread further apart so the eyes must move a farther distance and graduate from three to five images. Eye for detail includes four different stages of speed and discrimination, with each task consisting of 18 trials. Baseline speed begins by presenting each image for 500 ms and adjusts according to the participant’s accuracy (e.g. speeds up presentation if previous answer was correct). Total score is the presentation time in milliseconds of the final trial.

Hawk eye improves visual precision and working memory. Participants are required to identify the one image of a bird that is different from the other birds presented within their peripheral vision by clicking on the position with the mouse. The birds initially appear very distinct, close together and on a simple background, but as participants progress through the exercises the birds become more similar, are spread farther apart and the background becomes more complex. Hawk eye includes two different stages of speed and discrimination, with each task consisting of 35 trials. Baseline speed begins by presenting each image for 200 ms and adjusts according to the participant’s accuracy (e.g. speeds up presentation if previous answer was correct).Total score is the presentation time in milliseconds of the final trial.

#### Phase 2: Face specific visual training

The second phase of training involves visual training specifically relating to faces, including facial recognition and emotion recognition. All tasks will be administered through the Posit Science (Brain HQ) computer-training module. This phase will be administered in one-on-one face-to-face sessions with a provisional psychologist, who will instruct and support the participants. Specific visual training tasks were selected due to the relevance of visual face processing within BDD.

Face to face improves social cognition and visual processing speed, so participants can quickly and correctly interpret facial expressions. Participants are briefly shown a face and are then required to decide what expression they think it shows. They then choose the face showing the same expression from a set of faces by clicking with the mouse. As participants improve, more emotions are added, the intensity of the expression decreases, the faces flash up faster and the number of faces to choose from increases. The initial facial expressions include happy, sad, neutral, angry and surprised and then expand to include afraid, disgusted, proud, contemptuous, puzzled and embarrassed. Face to face includes six different stages of number of emotions, and intensity of emotional expression, with each task consisting of 20 trials. Baseline speed begins by presenting each image for 1000 ms and adjusts according to the participant’s accuracy (e.g. speeds up presentation if previous answer was correct). Total score is the presentation time in milliseconds of the final trial.

Recognition improves facial recognition and visual processing speed, so participants can quickly and accurately identify an individual’s face from a group of other faces. Participants are briefly shown a face and then required to identify which one they saw from a selection of faces, by clicking with the mouse. As participants improve, the angle of the face changes (straight on, 45° angle and profile), the number of faces to choose from increases, the faces flash up faster, and the faces become gender-matched. Recognition includes six different stages of number of faces, and characteristics of faces, with each task consisting of 20 trials. Baseline speed begins by presenting each image for 500 ms and adjusts according to the participant’s accuracy (e.g. speeds up presentation if previous answer was correct). The total score is the presentation time in milliseconds of the final trial.

#### Phase 3: Self-perception visual training

The third phase involves perceptual retraining using mirrors. This phase will be administered in one-on-one face-to-face sessions with a provisional psychologist, in accordance with the ‘Perceptual Retraining for Mirror Checking’ chapter from ‘Cognitive Behaviour Therapy for Body Dysmorphic Disorder: A Treatment Manual’ [83].

Mirror retraining helps to change how individuals look at themselves in the mirror, to a more mindful, nonjudgmental and holistic way, resulting in improved self-perception. This involves systematic exposure to mirror reflections while asking clients to take a different perspective than they typically do, e.g. see self holistically instead of focusing or avoiding hotspots. The purpose is to help clients gradually feel less anxious each time they look in a mirror, by changing the focus of their perception. Each exercise should take around 10 min. The provisional psychologist administering the visual training program has received specialised training in CBT for BDD, including perceptual mirror retraining.

### Sample size

As this is a pilot study, sample size calculations were not considered appropriate. The authors aimed for 20 participants as it was deemed that this sample size would be sufficient in assessing the practicalities of recruitment, intervention delivery and attrition in a clinical population. This sample size is sufficient in exploring qualitative themes of acceptability and reflects quantitative measures of clinical outcomes within this population.

### Analysis

The feasibility outcomes of recruitment rate, retention rate, adverse events, compliance and acceptability will be reported using a descriptive approach with 95% confidence intervals for any estimates. The qualitative data regarding acceptability will be subjected to, thematic analysis following Braun and Clarke’s (2006) guidelines. To prevent potential coding bias, the researcher conducting the semi-structured interview will not participate in the coding. The final coding structure will be validated with the remaining members of the research team.

Clinical outcomes will be reported as means, standard deviations and ranges for all outcome measures at T1–T4, with between-group differences analysed using a repeated measures analysis of variance (MANOVA). Due to the underpowered nature of the pilot trial, these results will focus on point estimates and associated 95% confidence intervals as opposed to significance testing, to explore potential efficacy of the program. Missing data will be dealt with by adopting appropriate imputation procedures which will include mean or last observation carried forward.

### Research governance and ethics

The trial has been approved by Swinburne University Human Research Ethics Committee (SUHREC; project 2017/333) and is being administered at Swinburne University. All participants provide full informed written consent prior to completing the initial assessment. As described in the information statement and consent form, any concerns or adverse events will be discussed in supervision and reported to the Swinburne University Human Research Ethics Committee (SUHREC) within 24 h. Participants will also be offered contact details of specialist clinicians if they are not receiving external support. This trial will be audited annually in accordance with SUHREC procedures. All participant data will be stored securely, with study data being de-identified and accessible only by the investigators. Participants will be informed that only group results will be used for publishing purposes. Any amendments to the protocol will be approved by SUHREC and communicated to the Australian New Zealand Clinical Trials Registry (ANZCTR) and all relevant parties.

## Discussion

Despite the growing literature of visual processing abnormalities being a core feature of BDD, this is rarely a target of treatment [58]. Indeed, only one study to date has attempted to alter visual perception among this population, with limited but promising findings [39]. Due to the poor long-term treatment outcomes and high relapse rates reported among BDD, there has been overwhelming support for a visual training program to address the often-neglected area of visual perception. As such, this pilot study aims to evaluate the feasibility and potential efficacy of a visual training program to remediate visual abnormalities and reduce symptom severity of individuals with BDD. It is expected that this program will show promising feasibility and acceptability outcomes, which may lead to the implementation of a definitive RCT which would be appropriately powered to assess efficacy compared to a control group. Additionally, clinical outcomes may demonstrate potential efficacy in terms of symptom severity, quality of life and eye movement data.

One practical issue of this study may involve recruitment numbers, based on the nature of BDD. As the disorder is such a secretive one, population estimates can be low, and those that have been diagnosed or identify with having BDD are often housebound or otherwise significantly impaired in daily functioning. As such, there may be some difficulty arranging for participants to attend a 10-week visual training program outside of their home. The authors propose that by framing this trial as a novel intervention study with the opportunity to learn new skills, individuals may see the potential benefits of participating.

Generalisability is another possible issue for this trial, as due to the often varied nature of BDD, the sample will only capture a small percentage of this population. In addition, individuals interested in participating in a treatment trial are likely to have certain traits which may distinguish them from the broader clinical population, such as increased motivation, and higher likelihood of concurrent psychological or psychiatric treatment. The authors acknowledge the bias implicit in a single-group open label pilot study, due to the lack of control group and randomisation. However, this methodology is believed necessary to initially explore the feasibility of such a program prior to a definitive RCT.

If these results demonstrate visual training is a feasible and acceptable method of remediating visual perception in BDD with the support of a future RCT, such programs may be used in combination with current treatments such as CBT. Based on the multi-faceted nature of BDD, it appears logical for treatment methods to target a combination of phenomenological features including abnormalities in visual perception; unhelpful beliefs about oneself, others and the world; distressing emotional responses; and time-consuming repetitive and obsessive behaviours.

## Trial status

This research study has been ethically approved, and recruitment has commenced. The first participant was recruited in April 2017, with expected completion in early 2018.

## Additional files

Additional file 1: Qualitative Analysis: Semi Structured Interview Guide. (DOCX 17 kb)

Additional file 2: A Visual Training Program Trial to Remediate Perceptual Abnormalities in Body Dysmorphic Disorder: In Between Sessions Questionnaire. (DOCX 15 kb)

## References

[1] American Psychiatric Association. Diagnostic and statistical manual of mental disorders (5th ed.). Washington, DC: Author; 2013.

[2] Veale D, Gledhill LJ, Christodoulou P, Hodsoll J (2016). Body dysmorphic disorder in different settings: a systematic review and estimated weighted prevalence. Body Image.

[3] Veale D, Boocock A, Gournay K, Dryden W, Shah F, Willson R (1996). Body dysmorphic disorder. A survey of fifty cases. Br J Psychiatry J Ment Sci.

[4] Phillips KA, Didie ER, Menard W (2007). Clinical features and correlates of major depressive disorder in individuals with body dysmorphic disorder. J Affect Disord.

[5] Phillips KA, Menard W, Fay C, Weisberg R (2005). Demographic characteristics, phenomenology, comorbidity, and family history in 200 individuals with body dysmorphic disorder. Psychosomatics.

[6] Tukel R, Tihan AK, Ozturk N (2013). A comparison of comorbidity in body dysmorphic disorder and obsessive-compulsive disorder. Ann Clin Psychiatry.

[7] Phillips KA, Coles ME, Menard W, Yen S, Fay C, Weisberg RB (2005). Suicidal ideation and suicide attempts in body dysmorphic disorder. J Clin Psychiatry.

[8] Phillips KA (2007). Suicidality in body dysmorphic disorder. Primary Psychiatry.

[9] National Collaborating Centre for Mental H. National Institute for Health and Clinical Excellence: Guidance (2006). Obsessive-compulsive disorder: core interventions in the treatment of obsessive-compulsive disorder and body dysmorphic disorder.

[10] Phillipou A, Rossell SL, Wilding HE, Castle DJ (2016). Randomised controlled trials of psychological & pharmacological treatments for body dysmorphic disorder: a systematic review. Psychiatry Res.

[11] Harrison A, de la Cruz LF, Enander J, Radua J, Mataix-Cols D (2016). Cognitive-behavioral therapy for body dysmorphic disorder: a systematic review and meta-analysis of randomized controlled trials. Clin Psychol Rev.

[12] Krebs G, de La Cruz LF, Monzani B, Bowyer L, Anson M, Cadman J (2017). Long-term outcomes of cognitive-behavioral therapy for adolescent body dysmorphic disorder. Behav Ther.

[13] Veale D, Riley S (2001). Mirror, mirror on the wall, who is the ugliest of them all? The psychopathology of mirror gazing in body dysmorphic disorder. Behav Res Ther.

[14] Fang A, Wilhelm S (2015). Clinical features, cognitive biases, and treatment of body dysmorphic disorder. Annu Rev Clin Psychol.

[15] Kerwin L, Hovav S, Hellemann G, Feusner JD (2014). Impairment in local and global processing and set-shifting in body dysmorphic disorder. J Psychiatr Res.

[16] Kimchi R (1992). Primacy of wholistic processing and global/local paradigm: a critical review. Psychol Bull.

[17] Love B, Rouder J, Wisniewski E (1999). A structural account of global and local processing. Cogn Psychol.

[18] Feusner JD, Moller H, Altstein L, Sugar C, Bookheimer S, Yoon J (2010). Inverted face processing in body dysmorphic disorder. J Psychiatr Res.

[19] Toh WL, Castle DJ, Rossell SL (2017). Face and object perception in body dysmorphic disorder versus obsessive-compulsive disorder: the Mooney Faces Task. J Int Neuropsychol Soc.

[20] Jefferies K, Laws KR, Fineberg NA (2012). Superior face recognition in body dysmorphic disorder. J Obsessive-Compulsive Relat Disord.

[21] Grocholewski A, Kliem S, Heinrichs N (2012). Selective attention to imagined facial ugliness is specific to body dysmorphic disorder. Body Image.

[22] Toh WL, Castle DJ, Rossell SL (2015). Facial affect recognition in body dysmorphic disorder versus obsessive-compulsive disorder: an eye-tracking study. J Anxiety Disord.

[23] Toh WL, Castle DJ, Rossell SL (2017). How individuals with body dysmorphic disorder (BDD) process their own face: a quantitative and qualitative investigation based on an eye-tracking paradigm. Cogn Neuropsychiatry.

[24] Phillipou A, Rossell SL, Castle DJ, Gurvich C, Abel LA (2014). Square wave jerks and anxiety as distinctive biomarkers for anorexia nervosa. Invest Ophthalmol Vis Sci.

[25] Andrea P, Susan Lee R, Caroline G, Matthew Edward H, David Jonathan C, Richard Grant N (2016). Saccadic eye movements in anorexia nervosa. PLoS One.

[26] Benson PJ, Beedie SA, Shephard E, Giegling I, Rujescu D, St. Clair D (2012). Simple viewing tests can detect eye movement abnormalities that distinguish schizophrenia cases from controls with exceptional accuracy. Biol Psychiatry.

[27] Morita K, Miura K, Fujimoto M, Yamamori H, Yasuda Y, Iwase M (2017). Eye movement as a biomarker of schizophrenia: using an integrated eye movement score. Psychiatry Clin Neurosci.

[28] Myles JB, Rossell SL, Phillipou A, Thomas E, Gurvich C (2017). Insights to the schizophrenia continuum: a systematic review of saccadic eye movements in schizotypy and biological relatives of schizophrenia patients. Neurosci Biobehav Rev.

[29] Feusner JD, Hembacher E, Moller H, Moody TD (2011). Abnormalities of object visual processing in body dysmorphic disorder. Psychol Med.

[30] Li W, Lai TM, Bohon C, Loo SK, McCurdy D, Strober M (2015). Anorexia nervosa and body dysmorphic disorder are associated with abnormalities in processing visual information. Psychol Med.

[31] Monzani B, Krebs G, Anson M, Veale D, Mataix-Cols D (2013). Holistic versus detailed visual processing in body dysmorphic disorder: testing the inversion, composite and global precedence effects. Psychiatry Res.

[32] Arienzo D, Leow A, Brown JA, Zhan L, GadElkarim J, Hovav S (2013). Abnormal brain network organization in body dysmorphic disorder. Neuropsychopharmacology.

[33] Yaryura-Tobias JA, Neziroglu F, Torres-Gallegos M (2002). Neuroanatomical correlates and somatosensorial disturbances in body dysmorphic disorder. CNS Spectr.

[34] Feusner JD, Townsend J, Bystritsky A, McKinley M, Moller H, Bookheimer S (2009). Regional brain volumes and symptom severity in body dysmorphic disorder. Psychiatry Res Neuroimaging.

[35] Madsen SK, Zai A, Pirnia T, Arienzo D, Zhan L, Moody TD (2015). Cortical thickness and brain volumetric analysis in body dysmorphic disorder. Psychiatry Res Neuroimaging.

[36] Buchanan B, Rossell S, Maller JJ, Toh WL, Brennan S, Castle D (2014). Regional brain volumes in body dysmorphic disorder compared to controls. Aust N Z J Psychiatry.

[37] Buchanan BG, Rossell SL, Maller JJ, Toh WL, Brennan S, Castle DJ (2013). Brain connectivity in body dysmorphic disorder compared with controls: a diffusion tensor imaging study. Psychol Med.

[38] Li W, Lai TM, Loo SK, Strober M, Mohammad-Rezazadeh I, Khalsa S, et al. Aberrant early visual neural activity and brain-behavior relationships in anorexia nervosa and body dysmorphic disorder. Front Hum Neurosci. 2015;9. 10.3389/fnhum.2015.00301. 10.3389/fnhum.2015.00301 PMC4451358 26082703

[39] Buhlmann U, Gleiss MJ, Rupf L, Zschenderlein K, Kathmann N (2011). Modifying emotion recognition deficits in body dysmorphic disorder: an experimental investigation. Depress Anxiety.

[40] Buhlmann U, Etcoff NL, Wilhelm S (2006). Emotion recognition bias for contempt and anger in body dysmorphic disorder. J Psychiatr Res.

[41] Buhlmann U, McNally RJ, Etcoff NL, Tuschen-Caffier B, Wilhelm S. Emotion recognition deficits in body dysmorphic disorder. J Psychiatr Res. 2004;38:201–6. 10.1016/S0022-3956(03)00107-9. 10.1016/s0022-3956(03)00107-9 14757335

[42] Feusner JD, Bystritsky A, Hellemann G, Bookheimer S (2010). Impaired identity recognition of faces with emotional expressions in body dysmorphic disorder. Psychiatry Res.

[43] Baldock E, Anson M, Veale D (2012). The stopping criteria for mirror-gazing in body dysmorphic disorder. Br J Clin Psychol.

[44] Windheim K, Veale D, Anson M (2011). Mirror gazing in body dysmorphic disorder and healthy controls: effects of duration of gazing. Behav Res Ther.

[45] Ball K, Berch DB, Helmers KF, Jobe JB, Leveck MD, Marsiske M, et al. Effects of cognitive training interventions with older adults: a randomized controlled trial. JAMA. 2002;288:2271–81. 10.1001/jama.288.18.2271. 10.1001/jama.288.18.2271 PMC2916176 12425704

[46] Wolinsky FD, Vander Weg MW, Howren MB, Jones MP, Dotson MM (2013). A randomized controlled trial of cognitive training using a visual speed of processing intervention in middle aged and older adults. PLoS One.

[47] Hooker CI, Bruce L, Fisher M, Verosky SC, Miyakawa A, D'Esposito M (2013). The influence of combined cognitive plus social-cognitive training on amygdala response during face emotion recognition in schizophrenia. Psychiatry Res.

[48] O'Brien JL, Edwards JD, Maxfield ND, Peronto CL, Williams VA, Lister JJ (2013). Cognitive training and selective attention in the aging brain: an electrophysiological study. Clin Neurophysiol.

[49] Popov T, Rockstroh B, Weisz N, Elbert T, Miller GA (2012). Adjusting brain dynamics in schizophrenia by means of perceptual and cognitive training. PloS one.

[50] Wolinsky FD, Mahncke HW, Weg MWV, Martin R, Unverzagt FW, Ball KK (2009). The ACTIVE cognitive training interventions and the onset of and recovery from suspected clinical depression. J Gerontol Ser B Psychol Sci Soc Sci.

[51] Ahmed AO, Hunter KM, Goodrum NM, Batten NJ, Birgenheir D, Hardison E (2015). A randomized study of cognitive remediation for forensic and mental health patients with schizophrenia. J Psychiatr Res.

[52] Edwards JD, Hauser RA, O'Connor ML, Valdés EG, Zesiewicz TA, Uc EY (2013). Randomized trial of cognitive speed of processing training in Parkinson disease. Neurology.

[53] Van Vleet TM, Hoang-duc AK, DeGutis J, Robertson LC (2011). Modulation of non-spatial attention and the global/local processing bias. Neuropsychologia.

[54] Van Vleet TM, DeGutis JM (2013). Cross-training in hemispatial neglect: auditory sustained attention training ameliorates visual attention deficits. Cortex.

[55] Mishra J, Merzenich MM, Sagar R (2013). Accessible online neuroplasticity-targeted training for children with ADHD. Child Adolesc Psychiatry Ment Health.

[56] Smith GE, Housen P, Yaffe K, Ruff R, Kennison RF, Mahncke HW (2009). A cognitive training program based on principles of brain plasticity: results from the Improvement in Memory with Plasticity-based Adaptive Cognitive Training (IMPACT) study. J Am Geriatr Soc.

[57] Anonymous (2015). Cognitive aging: progress in understanding and opportunities for action. Mil Med.

[58] Beilharz F, Castle DJ, Grace S, Rossell SL (2017). A systematic review of visual processing and associated treatments in body dysmorphic disorder.

[59] Eldridge SM, Chan CL, Campbell MJ, Bond CM, Hopewell S, Thabane L, et al. CONSORT 2010 statement: extension to randomised pilot and feasibility trials. Pilot Feasibility Stud. 2016;2. 10.1186/s40814-016-0105-8. 10.1186/s40814-016-0105-8 PMC5154046 27965879

[60] Hendriksen M, Van R, Peen J, Oudejans S, Schoevers R, Dekker J (2010). Psychometric properties of the Helping Alliance Questionnaire-I in psychodynamic psychotherapy for major depression. Psychother Res.

[61] De Weert-Van Oene GH, De Jong CAJ, Jörg F, Schrijvers GJP (1999). Measurements, instruments, scales, and tests: the Helping Alliance Questionnaire: psychometric properties in patients with substance dependence. Subst Use Misuse.

[62] Sheehan DV, Lecrubier Y, Sheehan KH, Amorim P, Janavs J, Weiller E (1998). The Mini-International Neuropsychiatric Interview (M.I.N.I.): the development and validation of a structured diagnostic psychiatric interview for DSM-IV and ICD-10. J Clin Psychiatry.

[63] Phillips KA (1996). The broken mirror: understanding and treating body dysmorphic disorder.

[64] Lecrubier Y, Sheehan DV, Weiller E, Amorim P, Bonora I, Harnett Sheehan K (1997). The MINI International Neuropsychiatric Interview (MINI). A short diagnostic structured interview: reliability and validity according to the CIDI. Eur Psychiatry.

[65] Phillips KA, Hollander E, Rasmussen SA, Aronowitz BR, DeCaria C, Goodman WK (1997). A severity rating scale for body dysmorphic disorder: development, reliability, and validity of a modified version of the Yale-Brown Obsessive Compulsive Scale. Psychopharmacol Bull.

[66] Phillips KA, Hart AS, Menard W (2014). Psychometric evaluation of the Yale–Brown Obsessive-Compulsive Scale Modified for Body Dysmorphic Disorder (BDD-YBOCS). J Obsessive-Compulsive Relat Disord.

[67] Oosthuizen P, Lambert T, Castle DJ (1998). Dysmorphic concern: prevalence and associations with clinical variables. Aust N Z J Psychiatry.

[68] Mancuso SG, Knoesen NP, Castle DJ (2010). The dysmorphic concern questionnaire: a screening measure for body dysmorphic disorder. Aust N Z J Psychiatry.

[69] Jorgensen L, Castle D, Roberts C, Groth-Marnat G (2001). A clinical validation of the dysmorphic concern questionnaire. Aust N Z J Psychiatry.

[70] Brohede S, Wingren G, Wijma B, Wijma K (2013). Validation of the Body Dysmorphic Disorder Questionnaire in a community sample of Swedish women. Psychiatry Res.

[71] Lovibond SH (1995). Manual for the depression anxiety stress scales. Second edition.. ed. Lovibond PFa, Psychology Foundation of Australia issuing b, editors.

[72] Antony MM, Bieling PJ, Cox BJ, Enns MW, Swinson RP (1998). Psychometric properties of the 42-Item and 21-Item Versions of the Depression Anxiety Stress Scales in clinical groups and a community sample. Psychol Assess.

[73] Mattick RP, Clarke JC (1998). Development and validation of measures of social phobia scrutiny fear and social interaction anxiety. Behav Res Ther.

[74] Fergus TA, Valentiner DP, Kim HS, McGrath PB (2014). The Social Interaction Anxiety Scale (SIAS) and the Social Phobia Scale (SPS): a comparison of two short-form versions. Psychol Assess.

[75] Avalos L, Tylka TL, Wood-Barcalow N (2005). The Body Appreciation Scale: development and psychometric evaluation. Body Image.

[76] Carson SH, Peterson JB, Higgins DM (2005). Reliability, validity, and factor structure of the Creative Achievement Questionnaire. Creat Res J.

[77] Gratz K, Roemer L (2004). Multidimensional assessment of emotion regulation and dysregulation: development, factor structure, and initial validation of the difficulties in emotion regulation scale. J Psychopathol Behav Assess.

[78] Hallion LS, Steinman SA, Tolin DF, Diefenbach GJ (2018). Psychometric properties of the Difficulties in Emotion Regulation Scale (DERS) and its short forms in adults with emotional disorders. Front Psychol.

[79] Hawthorne G, Richardson J, Osborne R (1999). The Assessment of Quality of Life (AQoL) instrument: a psychometric measure of health-related quality of life. Qual Life Res.

[80] Hawthorne G, Richardson J, Day NA (2001). A comparison of the Assessment of Quality of Life (AQoL) with four other generic utility instruments. Ann Med.

[81] Mehling WE, Price C, Daubenmier JJ, Acree M, Bartmess E, Stewart A (2012). The Multidimensional Assessment of Interoceptive Awareness (MAIA). PLoS One.

[82] Tavassoli T, Hoekstra RA, Baron-Cohen S. The Sensory Perception Quotient (SPQ): development and validation of a new sensory questionnaire for adults with and without autism. Molecular Autism. 2014;5. 10.1186/2040-2392-5-29. 10.1186/2040-2392-5-29 PMC4005907 24791196

[83] Wilhelm S, Phillips KA, Steketee G (2013). Cognitive-behavioral therapy for body dysmorphic disorder: a treatment manual.

