# Supplementary material for: Visual training program for body dysmorphic disorder: protocol for a novel intervention pilot and feasibility trial
Source: Pilot Feasibility Stud. 2018 Dec 21;4:189. doi: 10.1186/s40814-018-0384-3 (PMC6302469; doi:10.1186/s40814-018-0384-3)
Supplement: Supplementary file 1 — Qualitative Analysis: Semi Structured Interview Guide. (DOCX 17 kb) [file 40814_2018_384_MOESM1_ESM.docx]

Additional file 1

Qualitative Analysis: Semi Structured Interview Guide

1. **Introduction of participants**:

- Name & Age
- Diagnosis

1. **Overall experience:**

- What attracted you to the visual training program?
- What were your thoughts about the visual training program?
- What were your expectations of the program?

1. **General Benefits**

- What did the program do for you? General benefits?
- Which aspects of the training did you find most useful?
- Were there any aspects of the training you didn’t find useful?

1. **Stimulus:**

- What tasks did you like most or least?
- Were there any tasks that seemed unclear or irrelevant?
- What did you think about combining computer-based and therapist-lead tasks?

1. **Cognitive trainer:**

- What did you think was the role of the psychologist?
- How did you feel interacting with the psychologist?

1. **Structure:**

- Did you like the way the sessions were run?
- How did you find the time requirement?
- Is there anything you would have changed about the program?

Do you have anything else to add?
