# Supplementary material for: Visual training program for body dysmorphic disorder: protocol for a novel intervention pilot and feasibility trial
Source: Pilot Feasibility Stud. 2018 Dec 21;4:189. doi: 10.1186/s40814-018-0384-3 (PMC6302469; doi:10.1186/s40814-018-0384-3)
Supplement: Supplementary file 2 — A Visual Training Program Trial to Remediate Perceptual Abnormalities in Body Dysmorphic Disorder: In Between Sessions Questionnaire. (DOCX 15 kb) [file 40814_2018_384_MOESM2_ESM.docx]

Additional file 2

A Visual Training Program Trial to Remediate Perceptual Abnormalities in Body Dysmorphic Disorder: In Between Sessions Questionnaire

Participant ID:

Session Number:

Date:

1. Have you spent time over the last week thinking about the previous session? Yes/ No

1. How much time have you spent over the last week thinking about the previous session? ______________ minutes/hours
2. What have you been thinking about from the previous session? _______________________________________________________________________________________________________________________________________________________________________________________________________________

1. Have you practiced anything that you learned from the previous session? Yes/No

1. How much time have you spent over the last week practicing something learned from the previous session? ______________ minutes/hours

1. What have you been practicing that was learned from the previous session? _______________________________________________________________________________________________________________________________________________________________________________________________________________
